# Longitudinal Associations Between Peer Risk and Promotive Factors and Exposure to Community Firearm Violence Among Black Adolescents

**DOI:** 10.1002/jcop.70047

**Published:** 2025-10-10

**Authors:** Colleen S. Walsh, Terri N. Sullivan, Wendy L. Kliewer

**Affiliations:** ^1^ Department of Psychology Vanderbilt University Nashville Tennessee USA; ^2^ Department of Psychology Virginia Commonwealth University Richmond Virginia USA

**Keywords:** black adolescents, community firearm violence, longitudinal models, risk and promotive factors

## Abstract

Firearm violence is pervasive for Black early adolescents in disinvested urban communities. The current study used a subsample of a longitudinal school‐based dataset (*N* = 479, ages 10–17, 100% Black), which included middle school students from three urban communities who completed surveys at two time points (Fall, Spring). Cross‐lagged panel models were run to specify the directionality of relations between five firearm exposure items and four peer factors; multiple group models tested differences in paths by sex and by grade. Results indicated that community firearm violence exposure was associated with higher adolescent‐reported peer delinquency and peer support for aggression; peer support for nonviolence with less exposure over time. Significant cross‐lagged paths were identified for females and older adolescents, but less so for males and younger adolescents. Results may inform community‐relevant firearm violence prevention, intervention efforts for middle‐school adolescents living in disinvested communities with elevated community violence prevalence.

## 1 Introduction

Black adolescents in systematically disinvested urban communities bear the brunt of community firearm violence, suffering immediate (e.g., death, injury) and long‐term consequences (e.g., chronic psychological symptoms, financial challenges, disability) (Bancalari et al. 2022; Bottiani et al. 2021; Gastineau et al. 2021; Smith et al. 2020). Firearm violence in these settings is catalyzed by historic (e.g., HOLC maps and redlining) and present (e.g., housing policies) systemic racism and strategic community disinvestment (e.g., geographic isolation, resource deprivation) (Gastineau et al. 2021; Kravitz‐Wirtz et al. 2022; Nation et al. 2021). Though the literature has established risk and promotive factors related to exposure to community violence broadly, relatively few studies have specifically assessed bidirectional associations between potential risk and promotive factors and exposure to community firearm violence, despite its extreme consequences.

Early adolescence (10–14 years) is a developmental window of heightened vulnerability for Black adolescents in disinvested urban neighborhoods (Bancalari et al. 2022; Smith et al. 2020). This period is characterized by rapid physical, cognitive, and socioemotional changes as young people encounter new expectations and expand their social networks (Prinstein and Giletta 2016). During this time, peer groups become a primary context for socialization, and adolescents’ heightened sensitivity to peer approval, rejection, and modeling (Christie and Viner 2005; Dishion et al. 2004) can magnify the influence of peer risk and promotive factors on their exposure to contextual threats such as community firearm violence. Importantly, national epidemiological data highlight the developmental significance of this period: according to the Centers for Disease Control and Prevention (2024), firearm‐related injuries are the leading cause of death among U.S. adolescents, with marked increases in both fatal and nonfatal injuries during adolescence.

Because of the significant changes during early adolescence, assessing experiences over time may shed light on changes in behavior or outcomes (Faden et al. 2004). For example, the increasing time spent with peers and the influence of peer support and behaviors modeled on adolescents’ experiences suggest the need to consider grade differences in relations between potential peer‐related risk and promotive factors and firearm violence exposure (Nansel et al. 2013). Additionally, female and male adolescents have different social experiences due to a variety of biological (puberty) and societal (gender norms) influences, which makes the consideration of sex differences important (Prinstein and Giletta 2016). Though risk increases in early adolescence, this developmental period also provides opportunities for the development of behavior patterns that support positive youth development through engagement in educational, experiential learning, and recreational activities, as well as through prevention and intervention efforts. To date, peer factors have not been comprehensively evaluated in relation to exposure to community firearm violence. The current study identified potentially influential and malleable peer factors and the directionality of their relations with exposure to community firearm violence in a sample of Black early adolescents. In addition, multiple group analyses were conducted to determine differences in these associations by sex and grade.

### 1.1 Potential Peer Risk and Promotive Factors for Exposure to Community Firearm Violence for Black Early Adolescents

Prior literature and developmental theory underscore the particularly strong influence of peers during early adolescence, a developmental period marked by heightened sensitivity to peer approval and shifting social affiliations. The Social Development Model (SDM; Catalano et al. 2021), which integrates social learning theory (Bandura 1977) and social bond theory (Hirschi 1968), is especially relevant for understanding peer influences at this stage. The SDM posits dual socialization pathways: one characterized by risk processes (e.g., weak bonds with prosocial institutions and reinforcement for antisocial behavior) that increase the likelihood of maladaptive behaviors, and another characterized by promotive or protective processes (e.g., strong prosocial bonds, positive peer norms) that support healthy development.

Importantly, the salience and nature of peer influences vary by age and grade level. Younger adolescents in earlier grades may be more susceptible to peer modeling due to less mature cognitive control and identity formation, while older adolescents may experience intensified peer pressure related to delinquent behaviors as social hierarchies solidify (Coie and Kupersmidt 1983; Coie and Dodge 1983). These developmental dynamics suggest that the same peer behavior may exert different levels of influence across early and middle adolescence (Huston and Ripke 2006).

The violence literature has primarily emphasized peer‐related risk factors, such as delinquent peer behavior, co‐offending, and peer pressure to engage in violence, linking these influences to outcomes like gun carrying (Oliphant et al. 2019), firearm violence (Schmidt et al. 2019), and exposure to community violence (Lambert et al. 2005, 2013; Salzinger et al. 2006). However, these studies often focus on older adolescents or do not fully account for developmental variation within adolescence. To advance the field, it is critical to investigate promotive peer factors (e.g., peers who model prosocial coping, emotional regulation, or avoidance of violence) and to consider how these factors may differentially impact youth at distinct developmental stages, especially in disinvested urban contexts where early exposure is common and potentially cumulative.

#### 1.1.1 Delinquent and Prosocial Peers

The extant literature on peer affiliation and its influence on adolescent violence and firearm outcomes has primarily focused on delinquent peer behavior, leaving prosocial peer behavior greatly under‐considered. Adolescents with delinquent peers are at increased risk for aggression, exposure to community violence, and firearm‐related behavior. Black male adolescents have higher rates of both delinquent behavior and violence exposure due to predatory criminalization and institutional bias, which exacerbate negative outcomes (Foell et al. 2021).

Peers who engage in prosocial behavior, defined as intentional actions that support others (e.g., sharing, helping; Eisenberg et al. 2006), promote adolescents’ own prosocial engagement and are negatively associated with problem behaviors (Farrell et al. 2017; Padilla‐Walker et al. 2018). This aligns with Ecological Systems Theory, which emphasizes the reinforcing effects of proximal relationships (Bronfenbrenner 1979). However, research on the potential protective role of prosocial peers in relation to firearm violence exposure among Black youth remains limited.

Developmental theory offers insight into when and how peers influence behavior. The SDM (Catalano et al. 2021), which integrates social learning and social bond theories, suggests that peer influences operate through both risk and promotive pathways. During early adolescence, sensitivity to peer approval is heightened, and peer modeling may be especially powerful for younger adolescents, while older youth may be more responsive to direct peer pressure. Additionally, sex differences may shape these processes, with some studies suggesting that female adolescents may be more influenced by messages promoting relational prosociality, while males may respond more to overt behavioral modeling (Coleman and Farrell 2021). Understanding such variation may help tailor interventions by grade and sex.

#### 1.1.2 Peer Support for Aggression and Nonviolence

Peer messages that promote aggression and nonviolence represent theoretically and empirically relevant peer processes that may function as risk and protective factors for exposure to community firearm violence among Black adolescents. Peers often become increasingly influential during early adolescence, at times surpassing the influence of parents and caregivers (Christie and Viner 2005; Dishion et al. 2004; Prinstein and Giletta 2016). The nature of peer responses, whether encouraging aggressive retaliation or supporting nonviolent conflict resolution, may shape adolescents’ behavioral strategies in high‐violence environments. Messages that reinforce aggression may normalize retaliatory behavior and escalate conflict, whereas messages supporting nonviolence may promote alternative responses that reduce the likelihood of future violence exposure or engagement.

Although few studies have examined these dynamics among Black adolescents, one qualitative study by Farrell et al. (2010b) provides important context. In interviews with predominantly Black (97%) urban adolescents, participants described distinct peer and family responses to conflict that either promoted fighting or encouraged nonviolent approaches. This distinction underscores the heterogeneity of peer influence in violence‐exposed communities and highlights the importance of assessing both risk‐enhancing and promotive peer processes. Similarly, in an evaluation of the universal violence prevention program Responding in Peaceful and Positive Ways (RIPP), implementation of peer mediation strategies among predominantly Black sixth‐grade students was associated with reductions in fighting and fight‐related injuries (Farrell et al. 2003). These findings suggest that peer‐promoted nonviolence may serve as a modifiable protective factor during early adolescence, a developmental period characterized by increased social sensitivity and peer conformity.

### 1.2 Current Study

This study extends prior work by examining how peer support for aggression and nonviolence relate to exposure to community firearm violence, with attention to differences by sex and grade across early to middle adolescence. Given the predominance of cross‐sectional designs in the existing literature and limited attention to bidirectional influences, the current study takes an exploratory approach to: (1) examine whether malleable peer factors predict changes in exposure to firearm violence over 6 months; (2) assess whether exposure to firearm violence predicts changes in peer factors during the same time frame; and (3) explore whether these associations differ by sex and grade. The study uses a longitudinal design with a sample of Black middle school students from urban communities with high rates of community violence. Although hypotheses are exploratory, identifying potential modifiable peer influences that operate differently across subgroups may inform the timing (e.g., middle school years), targets (e.g., peer aggression vs. nonviolence), and populations (e.g., sex and grade) for future, developmentally tailored violence prevention efforts.

## 2 Methods

### 2.1 Study Design and Setting

Data for this project were collected as part of a larger project which evaluated the efficacy of the Olweus Bullying Prevention Program (OBPP; Farrell et al. 2018) in three urban middle schools located in a mid‐sized Southeastern U.S. city. The larger evaluation used a multiple baseline experimental design, with OBPP implementation beginning in one school during Year 2 of the study, the second school in Year 3, and the third school in Year 6. From February 2011 and June 2018 data were collected in 3‐month intervals corresponding to the Fall, Winter, Spring, and Summer of each school year, apart from the Fall of Years 1 and 6 and Summer of Year 8. The schools and communities were selected based on their high rates of truancy and high burden of violence exposure, respectively. A total of 74%–100% of students enrolled in the selected schools lived in lower socioeconomic status households, as indicated by rates of eligibility for federal free and reduced lunch programs.

### 2.2 Procedure

All procedures were approved by the university Institutional Review Board (IRB). Student participation was voluntary, and students could discontinue or limit participation at any time. Written parental consent and adolescent assent was obtained before data collection. During assessment, participants completed self‐administered audio‐assisted measures on computers, allowing them to read and hear the questions. Participants completed measures at school during the academic year and at their homes during the summer. Informed permission and consent from a parent or guardian and student assent were obtained from approximately 80% of eligible participants.

### 2.3 Missing by Design

Though data were collected four times each year individual students were randomly assigned to complete two of the four assessments each year of participation. Missingness by design is a way to effectively handle longitudinal data collection, as it can be used to reduce participant fatigue and testing effects that could occur if each student completed all four assessment waves in a year, while also providing a large sample. This design also allows for a pattern of data missing completely at random (MCAR), or planned missingness, which does not affect the precision of parameter estimates (Brown et al. 2000) and can lead to a higher quality of data (Little 2013). Accordingly, we examined study variables across a 6‐month interval (Fall to Spring), as this provided the most complete and reliable data given the missing by design approach, and it aligns with developmental theory emphasizing that meaningful socioemotional and behavioral changes often unfold across the school year during early adolescence.

### 2.4 Participants

A subsample of the greater longitudinal school‐based dataset (*N* = 479, ages 10–17, 100% Black) were used for the current study, which includes a sample of 6th (35%), 7th (32%), and 8th (34%) grade students at two time points (Fall and Spring). Participants were adolescents (*M*_*age*_ = 12.2 years). The sample was 52% of participants were female, 48% were male, though these were the only two response options for sex. In the larger study sample, 79% of participants identified their race as Black (including 6% of the overall sample who endorsed Black as well as one or more other racial identities), 6% identified as White, and 3% endorsed other racial identities. Additionally, 12% of participants, most of whom (79%) identified their ethnicity as Hispanic/Latino/a, did not endorse any race. The greater study sample of 2755 students is representative of students attending the sampled middle schools during the 8 years of the study.

### 2.5 Measures

#### 2.5.1 Exposure to Community Firearm Violence

The items used to examine exposure to community firearm violence are derived from the 20‐item adapted version of the Survey of Children's Exposure to Community Violence scale (Richters and Saltzman 1990). This scale is an adolescent self‐report measure used to assess the frequency an adolescent has been victimized or witnessed violence and violence‐related activities in the past 3 months. From the original scale, one firearm victimization and four witnessing items were independently examined in relation to the peer factors in this study. The victimization item was “actually been shot at with a gun?;” Witnessing items included “actually seen someone carrying a gun or knife;” “heard gunfire outside when you were in or near your home,” “heard gunfire outside when you were in or near your school building;” and “seen someone else be shot with a gun.” Items were prefaced with the stem “In the last 3 months, how many times have these things happened to you…” and rated on a 6‐point scale from 0 = *never* to 6 = *20 or more times*.

#### 2.5.2 Peer Prosocial and Delinquent Behaviors

The 17‐item adolescent self‐report Peer Behavior Scale (Farrell et al. 2017) included two subscales that indicate adolescents’ perception of their friends’ delinquent (10‐items) and prosocial (7‐items) behavior. Participants were first asked to indicate the number of close friends they had (i.e., “friends who you see more than once a week and that you like doing things with”). The 17 subsequent items were preceded with the stem: “Now, we want to ask you about the behavior of these close friends. We want to know how many of them, as far as you know, have done any of these things in the last 3 months.” For peer delinquent behavior, adolescents indicated how many of their friends have been involved in deviant activities (e.g., aggression/violence, substance use, and delinquency). An example item for peer delinquent behavior is “Skipped school without an excuse?” For prosocial behavior adolescents indicated how many of their friends have been involved in behaviors that are theorized to have a positive relation to adolescent adjustment outcomes and a negative relation to aggression (e.g., positive youth development activities, altruistic behavior, attempts to solve problems peacefully). An example item for peer prosocial behavior was “Helped people without expecting something back?” All items are rated on a 4‐point scale, ranging from 1 = *none of them* to 4 = *all of them*. Farrell et al. (2017) found support for the structure of the scale, with strong measurement invariance across gender, grades, settings, time, and intervention conditions. The measure demonstrated concurrent validity with adolescents’ problem behavior and prosocial behavior (Farrell et al. 2017). The alpha coefficient for the friends’ delinquent and prosocial behavior subscales were 0.77 and 0.82, respectively.

#### 2.5.3 Peer Support for Aggression and Nonviolence

The Peer Support for Aggression and Nonviolence Scale (Farrell et al. 2010a, 2010b) assesses adolescents’ expectation of their peers’ reaction to various ways the adolescents might respond to a challenging situation. The measure included six scenarios that describe a challenging situation (e.g., “Imagine you see two people about to start a fight”). All scenarios were followed by two response types: an aggressive response (e.g., “What would your friends think if you *cheered on a fight?*) and a non‐violent response (e.g., What would your friends think if you *went to get an adult?*). For each response, adolescents were asked how they think their friends would react if they made each response. Answer choices include a positive peer reaction (i.e., They would think that I did the right thing), a neutral reaction (i.e., They would not care), and a negative reaction (i.e., They would think I was being a punk). The measure has two distinct subscales: Perceived Support for Aggression and Perceived Support for Nonviolent Behavior. Response options included response choices represent: 1 = *positive peer reaction/support*, −1 = *negative peer reaction/support*, 0 = *neutral peer reaction/no support*. Scores represent the mean across items. Internal consistency for the Peer Support for Aggression subscale was 0.62 and 0.77 for the Support for Nonviolent Behavior subscale.

#### 2.5.4 Covariates

Sex from school records (i.e., biological sex, not gender identity) was dummy‐coded and incorporated into final models for each aim. Grade was dummy‐coded and included as a covariate (6th vs. 7th and 8th graders; 8th vs. 6th and 7th graders). Household demographic variables included living in a one‐caregiver household (0 = lives in a one‐caregiver household, 1 = *all other caregiver situations in the household*), two‐caregiver household (0 = *lives in a two‐caregiver household*, 1 = *all other caregiver situations in the household*), and presence of a father figure in the household (0 = no father figure present, 1 = *yes, a father figure is present*) at T1 and T2. Intervention status was also dummy‐coded and included as a covariate. “Intervention status” indicates students who were (1) or were not (0) enrolled in a school receiving the school‐based universal OBPP intervention at the time of data collection.

### 2.6 Analytic Approach

Data were cleaned in IBM SPSS version 28 (2021), and primary analyses were conducted in Mplus version 8.1 (Muthén and Muthén 2012), using maximum likelihood estimation with robust standard errors (MLR) and full information maximum likelihood (FIML), which uses all available observed responses to address missing data (Wang and Wang 2012). Sandwich estimators (i.e., TYPE = COMPLEX) were used to account for non‐independence resulting from students being clustered by cohort and school. Participant responses to items that were missing, involved multiple answers, were not asked, were skipped, were not applicable, were out of range, or were not included in the wave were coded as missing.

Relations between variables were tested using comprehensive cross‐lagged panel models that simultaneously accounted for stability paths and cross‐time associations between peer risk and promotive factors and exposure to community firearm violence. Each model was run separately for each of the five community firearm violence exposure items. This modeling strategy allowed us to test: (1) autoregressive effects for each peer and exposure variable from Time 1 (T1) to Time 2 (T2), (2) the effects of T1 peer variables on T2 firearm violence exposure, and (3) the effects of T1 firearm violence exposure on T2 peer variables, while controlling for their respective stabilities.

Covariates were selected a priori based on theoretical relevance, empirical evidence, and availability at baseline. We included variables that were potential confounders, i.e., factors associated with both the exposure and outcome but not on the causal pathway. This approach aimed to minimize bias without over adjusting for variables that may mediate the relationship of interest. We did not include baseline measures of mental health, substance use, or prior violence involvement, as these may represent intermediate variables rather than true confounders.

While we considered aggregating peer factors and outcome variables for model parsimony, we retained disaggregated models to preserve theoretically and developmentally meaningful distinctions. Specifically, the peer variables, delinquent peer behavior, prosocial peer behavior, peer support for aggression, and peer support for nonviolence, represent distinct constructs grounded in the SDM and Ecological Systems Theory. These theories emphasize unique socialization pathways that may differentially shape youth behavior across developmental stages. Similarly, we modeled each firearm violence exposure item separately (e.g., witnessing vs. victimization), as prior research suggests these experiences may have distinct psychological and behavioral consequences, particularly in high‐violence urban contexts.

We recognize that this analytic approach increases model complexity. However, we conducted tests of multicollinearity among peer predictors and exposure outcomes and found no indication of problematic collinearity (variance inflation factors < 2). We also revised the manuscript to clarify our analytic strategy and rationale for the modeling decisions, and to explicitly acknowledge the tradeoffs between model complexity and interpretability.

Differences in the strength of cross‐lagged associations by sex and grade were tested using multiple group analyses. For sex, we compared an unconstrained model (where path coefficients varied by sex) with a constrained model (where paths were fixed to be equal across sex). The same approach was used to examine differences by grade (comparing 6th vs. 7/8th grade youth, and 8th vs. 6/7th grade youth).

Model fit was assessed using the Chi‐square statistic (*χ*²), the Comparative Fit Index (CFI), and the Root Mean Square Error of Approximation (RMSEA). CFI values ≥ 0.90 (Bentler 1992) and RMSEA values ≤ 0.08 (Browne and Cudeck 1993) were interpreted as indicating adequate model fit. Differences between constrained and unconstrained models were evaluated using changes in CFI and RMSEA, the *χ*² difference test, and the Bayesian Information Criterion (BIC). Finally, the delta method (MacKinnon 2008) was used to test the significance of path differences by sex and grade. Where unconstrained models were preferred, follow‐up analyses isolated individual paths to determine which differed across groups.

Finally, a sensitivity power analysis using G*Power version 3.1.9.7 (Faul et al. 2009) indicated that our sample size of 479 was sufficiently powered (i.e., ≥ 0.80) to detect small‐to‐medium effects. Specifically, for two independent groups of approximately equal size (*n*_*₁*_ = 239, *n*_*₂*_ = 240), the design provided 80% power to detect effects of *d* ≥ 0.26 (90% power: *d* ≥ 0.30). For one‐sample or paired‐sample tests, the study was sensitive to effects as small as *d* ≥ 0.13 at 80% power (90% power: *d* ≥ 0.15). Similarly, for bivariate correlations, the sample allowed detection of effects of *r* ≥ 0.13 with 80% power (90% power: *r* ≥ 0.15). Thus, the study was adequately powered to detect small‐to‐moderate effects, but less well powered to detect very small effects.

## 3 Results

Descriptive statistics, including means and standard deviations, were examined for each study variable (see Table 1), and correlations among the measures and across waves are reported in Supporting Information S1: Table S1. To address highly skewed and kurtotic distributions, several variables were log‐transformed, including each of the five exposure to community firearm violence items and friend's delinquent behavior. All Time 1 variables were significantly correlated with their corresponding Time 2 variables, indicating stability across time (see Supporting Information S1: Table S1). Covariates varied by model and were included only if they were significantly correlated with one or more primary study variables. Correlations among predictors at T1 were included in the model specification. Across the five models, all T1 variables (peer risk and promotive factors and exposure item) were significantly positively associated with the corresponding T2 variables indicating stability. Additionally, the treatment condition covariate was significantly positively related to increased likelihood of having peers who support aggressive responses to conflict in T1 for all models.

**Table 1:** Descriptives for all study variables (*N* = 479).

Variable	Time 1 (Fall)			Time 2 (Spring)		
	*N*	Mean (SD)	Range	*N*	Mean (SD) or % (*n*)	Range
**Exposure to community firearm violence items (log transformed)**						
“actually been shot at with a gun?”	479	0.02 (0.10)	0.00–0.78	479	0.02 (0.90)	0.00–0.78
“actually seen someone carrying a gun or knife”	479	0.14 (0.22)	0.00–0.78	479	0.12 (0.21)	0.00–0.78
“heard gunfire outside when you were in or near your home”	479	0.29 (0.30)	0.00–0.78	479	0.25 (0.26)	0.00–0.78
“heard gunfire outside when you were in or near your school building”	479	0.05 (0.20)	0.00–0.78	479	0.04 (0.14)	0.00–0.78
“seen someone else be shot with a gun”	479	0.08 (0.20)	0.00–0.78	479	0.10 (0.14)	0.00–0.78
**Potential per promotive and risk factors**						
Peer prosocial behavior	446	2.35 (0.80)	1–4	371	2.31 (0.85)	1–4
Peer delinquent behavior (log transformed)	446	1.12 (0.30)	0.99–0.62	374	1.11 (0.30)	0.99–2.92
Peer support for aggression	406	−0.12 (0.56)	−1–1	338	−0.10 (0.53)	−1–1
Peer support for nonviolence	408	0.20 (0.60)	−1–1	337	0.16 (0.60)	−1–1
**Covariates**						
Biological sex (male)	479	48.2 (231)	0–1	—	—	—
Intervention condition (intervention active at school)	479	74.3 (365)	0–1	—	—	—
Grade 6th	479	34.4 (165)	0–1	—	—	—
Grade 7th	479	32.8 (157)	0–1	—	—	—
Grade 8th	479	32.8 (157)	0–1	—	—	—
Two caregiver household	479	35.3% (169)	0–1	479	0–1	48.9% (234)
One caregiver household	479	56.6% (271)	0–1	479	0–1	31.1% (149)
Father figure present in household	479	45.1% (216)	0–1	479	0–1	38% (182)

Abbreviation: SD, standard deviation.

Following the initial analysis, multiple group models were run to determine whether each model differed by sex and grade. In the case of Models 1, 2, 3, and 5, all three comparison models indicated that the unconstrained models fit the data well and were favored over the constrained models based on the significant *χ* ^2^ difference tests. Though the BIC value was lower in the constrained models than the BIC value for the unconstrained models, the significant *χ* ^2^ difference test took precedence (see Table 2 for all model fit indexes). This suggested there were significant differences among study paths by sex and grade for these models. Details for Model 4 are included alongside the other results.

**Table 2:** Model fit statistics for risk and promotive peer factors and exposure to community firearm violence multiple group models by sex and grade.

	Sex (female vs. male)		6th grade vs. 7th/8th grade		8th grade vs. 6th/7th grade	
	Constrained	Unconstrained	Constrained	Unconstrained	Constrained	Unconstrained
**“been shot at with a gun”**						
*p*	0.000	0.055	0.000	0.025	0.001	0.055
*χ* ^2^ (df)	291.30 (186)	178.76 (150)	274.59 (186)	185.84 (150)	252.78 (186)	178.76 (150)
RMSEA	0.05	0.03	0.05	0.03	0.04	0.03
CFI	0.87	0.97	0.90	0.96	0.92	0.97
BIC	2268.30	2377.93	2513.42	2646.86	2529.68	2677.85
df difference		36		36		36
*χ* ^2^ test		112.54		88.75		74.02
Selected model		X		X		X
**“seen someone carrying a gun or knife”**						
*p*	0.000	0.074	0.000	0.016	0.000	0.088
*χ* ^2^ (df)	258.34 (167)	156.46 (132)	259.81 (167)	169.35 (132)	242.14 (167)	154.50 (132)
RMSEA	0.05	0.03	0.05	0.03	0.04	0.03
CFI	0.89	0.97	0.89	0.95	0.91	0.97
BIC	4064.38	4178.31	4101.34	4226.79	4092.23	4220.60
df difference		35		35		35
*χ* ^2^ test		101.88		90.46		87.64
Selected model		X		X		X
**“heard gunfire in or near your home”**						
*p*	0.000	0.084	0.000	0.017	0.001	0.148
*χ* ^2^ (df)	240.82 (147)	133.16 (112)	224.65 (147)	146.02 (112)	210.34 (147)	127.67 (112)
RMSEA	0.05	0.03	0.05	0.04	0.04	0.02
CFI	0.89	0.98	0.91	0.96	0.92	0.98
BIC	4429.62	4537.97	4449.98	4587.36	4448.07	4581.41
df difference		35		35		35
*χ* ^2^ test		107.66		78.63		82.67
Selected model		X		X		X
**“heard gunfire in or near your school”**						
*p*	0.001	0.061	0.000	0.016	0.003	0.052
*χ* ^2^ (df)	232.50 (167)	157.96 (132)	241.71 (167)	169.20 (132)	220.62 (167)	159.45 (132)
RMSEA	0.04	0.03	0.04	0.03	0.04	0.03
CFI	0.91	0.96	0.90	0.95	0.93	0.96
BIC	3477.14	3618.61	3568.91	3721.41	3574.86	3729.70
df difference		35		35		35
*χ* ^2^ test		74.54		72.51		61.17
Selected model		X		X		X
**“seen someone be shot at”**						
*p*	0.000	0.009	0.000	0.003	0.001	0.079
*χ* ^2^ (df)	269.89 (167)	173.43 (132)	153.46 (167)	182.09 (132)	227.23 (167)	155.60 (132)
RMSEA	0.05	0.04	0.05	0.04	0.04	0.03
CFI	0.88	0.95	0.90	0.94	0.93	0.97
BIC	3475.94	3595.49	3483.44	3628.07	3512.33	3656.71
df difference		35		35		35
*χ* ^2^ test		96.46		28.63		71.63
Selected model		X	X			X

*Note:* β = regression coefficient, SE = standard error of parameter estimates, *χ* ^2^(df) = Chi‐square test of model fit (degrees of freedom), Critical *χ* ^2^ difference values at the *p* < 0.05 level by df: 30–> 43.77; 35–> 49.82; 40–> 55.76; 45–> 61.63.

Abbreviations: BIC, Bayesian Information Criterion; CHI, comparative fit index; RMSEA, root mean square error of approximation.

### 3.1 Model 1: Peer Risk and Promotive Factors and “Being Shot at With a Gun”

The first model included being shot at with a gun as the exposure variable, and peer risk and promotive factors. The model fit the data well *χ* ^2^ (75, *N* = 479) = 108.07, *p* = 0.008, RMSEA = 0.03, CFI = 0.96, SRMR = 0.05. Covariates in this model included T1 and T2 one‐ and two‐caregiver household, father figure present, and treatment condition. Cross‐lagged paths between peer delinquent behavior and adolescent‐report of being shot at with a gun were significantly positively associated with one another, indicating an increase at T2 after accounting for stability paths, other cross‐lagged paths, and covariates. This indicates that adolescents who have been shot at with a gun at T1 had an increased likelihood of having peers who engaged in delinquent behavior at T2, as well as adolescents who have peers who engage in more delinquency at T1 were more likely to report being shot at with a gun at T2. No other paths were significant (see Table 3).

**Table 3:** Model 1. Cross‐lagged path Model 1.

Effects	Original model		
	*β*	SE	*p*
**Path A, T1 peer factor on T2 peer factor**			
Peer delinquent behavior	0.41	0.05	< 0.001
Peer prosocial behavior	0.42	0.04	< 0.001
Peer support for aggression	0.51	0.04	< 0.001
Peer support for nonviolence	0.50	0.04	< 0.001
**Path B, T1 exposure on T2 exposure**			
Victimization “been shot at?”	0.38	0.05	< 0.001
**Path C, T1 exposure on T2 peer factor**			
“been shot at?” to peer delinquent behavior	0.18	0.05	0.001
“been shot at?” to peer prosocial behavior	0.06	0.05	0.24
“been shot at?” to peer support for aggression	0.08	0.05	0.10
“been shot at?” to peer support for nonviolence	0.01	0.05	0.83
**Path D, T1 peer factor on T2 exposure**			
Peer delinquent behavior to “been shot at?”	0.17	0.05	0.001
Peer prosocial behavior to “been shot at?”	−0.07	0.05	0.17
Peer support for aggression to “been shot at?”	−0.08	0.06	0.19
Peer support for nonviolence to “been shot at?”	−0.12	0.06	0.05
**Covariates**			
T2 2 caregiver household to T2 “been shot at?”	−0.003	0.10	0.98
T2 1 caregiver household to T2 “been shot at?”	−0.05	0.09	0.60
T2 father figure present to T2 “been shot at?”	0.02	0.06	0.71
T1 father figure present to T1 “been shot at?”	−0.07	0.04	0.10
T1 2 caregiver household to T1 peer support for aggression	−0.10	0.08	0.24
T1 1 caregiver household to T1 peer support for aggression	−0.04	0.08	0.64
Treatment condition to T1 peer support for aggression	0.08	0.04	0.03

*Note: N* = 479, β = regression coefficient, SE = standard error of parameter estimates, T1 = Time 1 (Fall), T2 = Time 2 (Spring). Unconstrained model data is reflective of the models before follow up analyses.

#### 3.1.1 “Being Shot at With a Gun” by Sex and Grade

In follow up analyses, results indicated males (*β* = 0.30, SE = 0.07, *p* < 0.001) but not females (*β* = 0.06, SE = 0.07, *p* = 0.76) and 6th and 7th graders (*β* = 0.19, SE = 0.06, *p* = 0.001) but not 8th graders (*β* = 0.11, SE = 0.09, *p* = 0.22) who reported more T1 peer delinquency reported higher rates of being shot at with a gun at T2. Female adolescents (*β* = 0.18, SE = 0.06, *p* = 0.003) but not male adolescents (*β* = 0.16, SE = 0.08, *p* = 0.051), 7th and 8th graders (*β* = 0.17, SE = 0.05, *p* = 0.004) but not 6th graders (*β* = 0.09, SE = 0.05, *p* = 0.41), and 6th and 7th graders (*β* = 0.21, SE = 0.06, *p* = 0.001) but not 8th graders (*β* = 0.19, SE = 0.10, *p* = 0.05) who reported being shot at with a gun at T1 was significantly related to a higher reported frequency of peer delinquency at T2. An additional post hoc analysis where a multiple group model compared 7th graders’ responses to 6th and 8th graders’ responses found that 7th graders (*β* = 0.10, SE = 0.07, *p* < 0.01) and not 6th and 8th graders (*β* = 0.10, SE = 0.08, *p* = 0.20) had a significant positive relation between being shot at and peer delinquent behavior 6 months later.

Uniquely, 8th graders (*β* = 0.26, SE = 0.05, *p* = 0.004) but not 6th and 7th graders (*β* = 0.05, SE = 0.05, *p* = 0.44) who reported being shot at with a gun at T1 was significantly related to a higher reported frequency of peers who support aggressive response to conflict at T2; 8th graders (*β* = −0.19, SE = 0.10, *p* = 0.045) but not 6th and 7th graders (*β* = −0.09, SE = 0.06, *p* = 0.18) reported high frequency of peers who supported nonviolent responses to conflict at T1 had lower likelihood of being shot at with a gun at T2; 8th graders (*β* = −0.20, SE = 0.08, *p* = 0.02) but not 6th and 7th graders (*β* = −0.03, SE = 0.06, *p* = 0.66) who reported high frequency of peers with prosocial behavior at T1 had lower likelihood of being shot at with a gun at T2.

### 3.2 Model 2: Peer Risk and Promotive Factors and “Seen Someone Carrying a Gun or Knife”

The second model included having seen someone carrying a gun or knife as the exposure variable, and peer risk and promotive factors. The model fit the data well *χ* ^2^ (66, *N* = 479) = 94.90, *p* = 0.01, RMSEA = 0.03, CFI = 0.96, SRMR = 0.05. Covariates in this model included T1 and T2 one‐ and two‐caregiver household, T2 father figure present, and treatment condition. Cross‐lagged paths between adolescent‐report of having seen someone carrying a gun or knife at T1 and higher reported frequency of peers who support aggressive responses to conflict at T2, and T1 report of peers who support nonviolent responses to conflict with lower reports of having seen someone carrying a gun or knife at T2 after accounting for stability paths, other cross‐lagged paths, and covariates. No other paths were significant (see Table 4).

**Table 4:** Model 2. All stability and cross‐lagged paths.

Effects	Original model		
	*β*	SE	*p*
**Path A, T1 peer factor on T2 peer factor**			
Peer delinquent behavior	0.29	0.05	< 0.001
Peer prosocial behavior	0.42	0.04	< 0.001
Peer support for aggression	0.48	0.04	< 0.001
Peer support for nonviolence	0.50	0.04	< 0.001
**Path B, T1 exposure on T2 exposure**			
Witnessing “seen someone carrying a gun or knife?”	0.44	0.04	< 0.001
**Path C, T1 exposure on T2 peer factor**			
“seen someone carrying a gun or knife?” to peer delinquent behavior	0.10	0.05	0.05
“seen someone carrying a gun or knife?”to peer prosocial behavior	−0.01	0.05	0.84
“seen someone carrying a gun or knife?” to peer support for aggression	0.15	0.05	0.002
“been shot at?” to peer support for nonviolence	0.000	0.05	0.10
**Path D, T1 peer factor on T2 exposure**			
Peer delinquent behavior to “seen someone carrying a gun or knife?”	0.05	0.05	0.31
Peer prosocial behavior to “seen someone carrying a gun or knife?”	−0.04	0.05	0.44
Peer support for aggression to “seen someone carrying a gun or knife?”	−0.03	0.06	0.65
Peer support for nonviolence to “seen someone carrying a gun or knife?”	−0.13	0.06	0.04
**Covariates**			
T2 2 caregiver household to T2 “seen someone carrying a gun or knife?”	0.06	0.11	0.61
T2 1 caregiver household to T2 “seen someone carrying a gun or knife?”	0.04	0.09	0.69
T2 father figure present to T2 “seen someone carrying a gun or knife?”	0.03	0.06	0.63
T1 2 caregiver household to T1 peer support for aggression	−0.11	0.08	0.20
T1 1 caregiver household to T1 peer support for aggression	−0.04	0.08	0.61
Treatment condition to T1 peer support for aggression	0.09	0.04	0.03

*Note: N* = 479, β = regression coefficient, SE = standard error of parameter estimates, T1 = Time 1 (Fall), T2 = Time 2 (Spring). Unconstrained model data is reflective of the models before follow up analyses.

#### 3.2.1 “Seen Someone Carrying a Gun or Knife” by Sex and Grade

In follow up analyses, results indicated that females (*β* = 0.16, SE = 0.07, *p* = 0.02) but not males (*β* = 0.06, SE = 0.07, *p* = 0.43) and 6th and 7th graders (*β* = 0.15, SE = 0.06, *p* = 0.01) but not 8th graders (*β* = 0.04, SE = 0.10, *p* = 0.66) who reported seeing someone carrying a gun or knife at T1 reported higher likelihood of having friends with delinquent behavior at T2. Females (*β* = 0.18, SE = 0.06, *p* = 0.003) but not males (*β* = 0.12, SE = 0.07, *p* = 0.08) and 7th and 8th graders (*β* = 0.17, SE = 0.06, *p* = 0.003) but not 6th graders (*β* = 0.14, SE = 0.08, *p* = 0.08) who reported seeing someone carrying a gun or knife at T1 reported higher peer support for aggressive responses to conflict at T2. For females (*β* = −0.16, SE = 0.08, *p* = 0.04) but not males (*β* = −0.10, SE = 0.08, *p* = 0.20), 6th graders (*β* = −0.18, SE = 0.09, *p* = 0.035) but not 7th and 8th graders (*β* = −0.11, SE = 0.07, *p* = 0.12), and 6th and 7th graders (*β* = −0.15, SE = 0.06, *p* = 0.03) but not 8th graders (*β* = −0.01, SE = 0.10, *p* = 0.61), participants who reported higher peer support for nonviolent response to conflict at T1 reported lower rates of seeing someone carrying a gun or knife at T2.

### 3.3 Model 3: Peer Risk and Promotive Factors and “Heard Gunfire Outside When in or Near Home”

The third model included being shot at with a gun as the exposure variable, and peer risk and promotive factors. The model fit the data well *χ* ^2^ (56, *N* = 479) = 85.57, *p* = 0.006, RMSEA = 0.03, CFI = 0.97, SRMR = 0.05. Covariates in this model included T1 and T2 one‐caregiver household, T1 two‐caregiver household, T1 father figure present, and treatment condition. There was only one significant and positive cross‐lagged path between adolescents who reported hearing gunfire outside when in or near home at T1 and higher reported frequency of peers with delinquent behavior at T2, after accounting for stability paths, other cross‐lagged paths, and covariates. No other paths were significant (see Table 5).

**Table 5:** Model 3. All stability and cross‐lagged paths.

Effects	Original model		
	*β*	SE	*p*
**Path A, T1 peer factor on T2 peer factor**			
Peer delinquent behavior	0.40	0.05	< 0.001
Peer prosocial behavior	0.42	0.04	< 0.001
Peer support for aggression	0.50	0.04	< 0.001
Peer support for nonviolence	0.50	0.04	< 0.001
**Path B, T1 exposure on T2 exposure**			
Witnessing “heard gunfire when in or near home?”	0.47	0.04	< 0.001
**Path C, T1 exposure on T2 peer factor**			
“heard gunfire when in or near home?” to peer delinquent behavior	0.11	0.05	0.02
“heard gunfire when in or near home?” to peer prosocial behavior	−0.01	0.05	0.86
“heard gunfire when in or near home?” to peer support for aggression	0.08	0.05	0.10
“heard gunfire when in or near home?” to peer support for nonviolence	0.03	0.05	0.49
**Path D, T1 peer factor on T2 exposure**			
Peer delinquent behavior to “heard gunfire when in or near home?”	0.04	0.05	0.46
Peer prosocial behavior to “heard gunfire when in or near home?”	0.004	0.05	0.93
Peer support for aggression to “heard gunfire when in or near home?”	0.08	0.06	0.20
Peer support for nonviolence to “heard gunfire when in or near home?”	−0.01	0.06	0.90
**Covariates**			
T1 1 caregiver household to T2 “heard gunfire when in or near home?”	−0.07	0.05	0.17
T2 1 caregiver household to T2 “heard gunfire when in or near home?”	−0.02	0.05	0.73
T1 father figure present to T1 “heard gunfire when in or near home?”	−0.05	0.04	0.28
T1 2 caregiver household to T1 peer support for aggression	−0.10	0.08	0.21
T1 1 caregiver household to T1 peer support for aggression	−0.04	0.08	0.66
Treatment condition to T1 peer support for aggression	0.08	0.04	0.03

*Note: N* = 479, β = regression coefficient, SE = standard error of parameter estimates, T1 = Time 1 (Fall), T2 = Time 2 (Spring). Unconstrained model data is reflective of the models before follow up analyses.

#### 3.3.1 “Heard Gunfire Outside When in or Near Home” by Sex and Grade

In follow up analyses, results indicated males (*β* = 0.17, SE = 0.07, *p* = 0.01) but not females (*β* = 0.04, SE = 0.07, *p* = 0.58), 6th graders (*β* = 0.29, SE = 0.08, *p* < 0.001) but not 7th and 8th graders (*β* = 0.03, SE = 0.06, *p* = 0.64), and 6th and 7th graders (*β* = 0.16, SE = 0.06, *p* = 0.01) but not 8th graders (*β* = 0.06, SE = 0.09, *p* = 0.48) who reported having heard gunfire outside when in or near home at T1 reported higher likelihood of having friends with delinquent behavior at T2. Additionally, females (*β* = 0.14, SE = 0.07, *p* = 0.045) but not males (*β* = −0.06, SE = 0.08, *p* = 0.94) and 8th graders (*β* = 0.21, SE = 0.09, *p* = 0.02) but not 6th and 7th graders (*β* = 0.05, SE = 0.07, *p* = 0.51) who reported higher peer support for aggressive responses to conflict at T1 had increased likelihood of having heard gunfire outside when in or near home at T2. Uniquely, 8th graders (*β* = 0.17, SE = 0.08, *p* = 0.03) but not 6th and 7th graders (*β* = 0.07, SE = 0.06, *p* = 0.25) who reported hearing gunfire outside while in or near home had an increased likelihood of having peers support aggressive responses to conflict at T2.

### 3.4 Model 4: Peer Risk and Promotive Factors and “Heard Gunfire Outside When in or Near School”

The fourth model included having heard gunfire outside when in or near school as the exposure variable, and peer risk and promotive factors. The model fit the data well *χ* ^2^ (66, *N* = 479) = 96.43, *p* = 0.009, RMSEA = 0.03, CFI = 0.96, SRMR = 0.05. Covariates in this model included T1 and T2 one‐ and two‐caregiver household, T2 father figure present, and treatment condition. There was only one significant and positive cross‐lagged path between adolescent reports of having heard gunfire outside when in or near school at T1 and higher reported frequency of peers with delinquent behavior at T2 after accounting for stability paths, other cross‐lagged paths, and covariates. No other paths were significant (see Table 6).

**Table 6:** Model 4. All stability and cross‐lagged paths.

Effects	Original model		
	*β*	SE	*p*
**Path A, T1 peer factor on T2 peer factor**			
Peer delinquent behavior	0.41	0.05	< 0.001
Peer prosocial behavior	0.42	0.04	< 0.001
Peer support for aggression	0.51	0.04	< 0.001
Peer support for nonviolence	0.49	0.04	< 0.001
**Path B, T1 exposure on T2 exposure**			
Witnessing “heard gunfire when in or near school?”	0.18	0.05	0.001
**Path C, T1 exposure on T2 peer factor**			
“heard gunfire when in or near school?” to peer delinquent behavior	0.11	0.05	0.03
“heard gunfire when in or near school?” to peer prosocial behavior	−0.04	0.05	0.39
“heard gunfire when in or near school?” to peer support for aggression	−0.002	0.05	0.96
“heard gunfire when in or near school?” to peer support for nonviolence	0.03	0.05	0.63
**Path D, T1 peer factor on T2 exposure**			
Peer delinquent behavior to “heard gunfire when in or near school?”	0.04	0.05	0.40
Peer prosocial behavior to “heard gunfire when in or near school?”	0.08	0.06	0.14
Peer support for aggression to “heard gunfire when in or near school?”	0.07	0.07	0.28
Peer support for nonviolence to “heard gunfire when in or near school?”	0.01	0.07	0.90
**Covariates**			
T2 2 caregiver household to T2 “heard gunfire when in or near school?”	−0.19	0.12	0.11
T2 1 caregiver household to T2 “heard gunfire when in or near school?”	−0.19	0.10	0.07
T2 father figure present to T2 “heard gunfire when in or near school?”	0.01	0.06	0.86
T1 2 caregiver household to T1 peer support for aggression	−0.11	0.08	0.19
T1 1 caregiver household to T1 peer support for aggression	−0.05	0.08	0.56
Treatment condition to T1 peer support for aggression	0.08	0.04	0.03

*Note: N* = 479, β = regression coefficient, SE = standard error of parameter estimates, T1 = Time 1 (Fall), T2 = Time 2 (Spring). Unconstrained model data is reflective of the models before follow up analyses.

#### 3.4.1 “Heard Gunfire Outside When in or Near School” by Sex and Grade

Multiple group models were run to determine whether Model 4 differed by sex and grade. In all three cases, the unconstrained models fit the data well and were favored over the constrained models based on the significant *χ* ^2^ difference tests. Though the BIC value was lower in the constrained models than the BIC value for the unconstrained models, the significant *χ* ^2^ difference test took precedence (see Table 2 for all model fit indexes). This suggested there were significant differences among study paths by sex and grade. However, only differences were found by path in the analyses testing differences between female and male adolescents, and 6th graders and 7th and 8th graders combined, but not 8th graders and 6th and 7th graders combined.

In follow up analyses, female youth (*β* = 0.17, SE = 0.06, *p* = 0.01) but not males (*β* = 0.07, SE = 0.07, *p* = 0.32) and 7th and 8th graders (*β* = 0.12, SE = 0.06, *p* = 0.04) but not 6th graders (*β* = 0.06, SE = 0.09, *p* = 0.49) who reported having heard gunfire outside when in or near school at T1 reported higher likelihood of having friends with delinquent behavior at T2. Additionally, 7th and 8th graders (*β* = 0.13, SE = 0.06, *p* = 0.03) but not 6th graders (*β* = −0.11, SE = 0.09, *p* = 0.20) who reported increased reports of hearing gunfire in or near school at T1 had increased likelihood of reporting having peers who support nonviolent responses to conflict at T2.

### 3.5 Model 5: Peer Risk and Promotive Factors and “See Someone Shot With a Gun”

The fifth model included having heard gunfire outside when in or near school as the exposure variable, and peer risk and promotive factors. The model fit the data well *χ* ^2^ (66, *N* = 479) = 101.11, *p* = 0.004, RMSEA = 0.03, CFI = 0.96, SRMR = 0.05. Covariates in this model included T1 and T2 one‐ and two‐caregiver household, T2 father figure present, and treatment condition. There was only one significant and positive cross‐lagged path between adolescent reports of having seen someone be shot with a gun at T1 and higher reported frequency of peers with delinquent behavior at T2 after accounting for stability paths, other cross‐lagged paths, and covariates. No other paths were significant (see Table 7).

**Table 7:** Model 5. All stability and cross‐lagged paths.

Effects	Original model		
	*β*	SE	*p*
**Path A, T1 peer factor on T2 peer factor**			
Peer delinquent behavior	0.39	0.05	< 0.001
Peer prosocial behavior	0.42	0.04	< 0.001
Peer support for aggression	0.50	0.04	< 0.001
Peer support for nonviolence	0.49	0.04	< 0.001
**Path B, T1 exposure on T2 exposure**			
Witnessing “seen someone be shot at?”	0.48	0.04	< 0.001
**Path C, T1 exposure on T2 peer factor**			
“seen someone be shot at?” to peer delinquent behavior	0.14	0.05	0.01
“seen someone be shot at?” to peer prosocial behavior	0.03	0.05	0.53
“seen someone be shot at?” to peer support for aggression	0.09	0.05	0.07
“seen someone be shot at?” to peer support for nonviolence	−0.004	0.05	0.93
**Path D, T1 peer factor on T2 exposure**			
Peer delinquent behavior to “seen someone be shot at?”	0.04	0.05	0.50
Peer prosocial behavior to “seen someone be shot at?”?”	0.01	0.05	0.80
Peer support for aggression to “seen someone be shot at?”	−0.04	0.06	0.52
Peer support for nonviolence to “seen someone be shot at?”	−0.001	0.06	0.99
**Covariates**			
T2 2 caregiver household to T2 “seen someone be shot at?”	0.01	0.10	0.91
T2 1 caregiver household to T2 “seen someone be shot at?”	−0.02	0.09	0.80
T2 father figure present to T2 “seen someone be shot at?”	0.05	0.06	0.39
T1 2 caregiver household to T1 peer support for aggression	−0.09	0.08	0.30
T1 1 caregiver household to T1 peer support for aggression	−0.02	0.08	0.80
Treatment condition to T1 peer support for aggression	0.08	0.04	0.04

*Note: N* = 479, β = regression coefficient, SE = standard error of parameter estimates, T1 = Time 1 (Fall), T2 = Time 2 (Spring). Unconstrained model data is reflective of the models before follow up analyses.

#### 3.5.1 “See Someone Shot With a Gun” by Sex and Grade

In follow up analyses, results indicated 7th and 8th graders (*β* = 0.13, SE = 0.06, *p* = 0.04) but not 6th graders (*β* = 0.12, SE = 0.09, *p* = 0.18) and 8th graders (*β* = 0.21, SE = 0.08, *p* = 0.008) but not 6th and 7th graders (*β* = 0.11, SE = 0.07, *p* = 0.10) who reported seeing someone be shot at with a gun in T1 reported increased likelihood of having peers who engaged in delinquent behavior in T2. Additionally, 7th and 8th graders (*β* = 0.13, SE = 0.06, *p* = 0.02) but not 6th graders (*β* = 0.04, SE = 0.08, *p* = 0.65) and 8th graders (*β* = 0.18, SE = 0.08, *p* = 0.02) but not 6th and 7th graders (*β* = 0.07, SE = 0.06, *p* = 0.25) who reported seeing someone shot at with a gun at T1 was significantly related to a higher reported frequency of peers who support aggressive response to conflict at T2. Uniquely, males (*β* = −0.16, SE = 0.07, *p* = 0.025) but not females (*β* = 0.07, SE = 0.07, *p* = 0.34) who reported having peers who supported aggressive responses to conflict at T1 were less likely to report having seen someone shot with a gun at T2; 7th and 8th graders (*β* = 0.12, SE = 0.06, *p* = 0.04) but not 6th graders (*β* = −0.14, SE = 0.06, *p* = 0.04) who reported having peers with delinquent behavior at T1 were more likely to have seen someone shot with a gun at T2.

For a summary of all cross‐lagged findings, see Supporting Information S1: Table S2.

## 4 Discussion

Firearm violence and injury disproportionately impacts Black early adolescents in economically marginalized, urban communities. Despite an uptick in funding and research on risk and promotive factors for community firearm violence and firearm behaviors (e.g., carrying, perpetration), major gaps remain in our understanding of the bidirectional longitudinal associations between specific peer factors (e.g., beliefs, messages, behaviors) and community firearm violence exposure. The current study tested (1) the degree to which malleable peer factors predicted changes in exposure to community firearm violence over 6 months, (2) the extent to which exposure to community firearm violence predicted changes in the peer factors over 6 months, and (3) the extent to which the strength of these relations varied by sex and grade in a sample of primarily Black middle school students in disinvested urban communities with high rates of community violence. Understanding the interplay of community firearm violence exposure (e.g., hearing gunfire, seeing others be injured by a gun, being shot with a gun) and aspects of early adolescents’ social relationships with peers may broaden our understanding of specific social behaviors and interactions, messages, and beliefs that change the likelihood of being exposed to firearm violence in their communities, and the consequences of firearm violence exposure on adolescents’ peer relationships.

### 4.1 Longitudinal Associations Between Community Firearm Violence Exposure and Peer Risk and Promotive Factors

Across the five primary models, all exposure, risk, and promotive factors at Time 1 were significantly and positively associated with themselves at Time 2. The significant stability paths are conceptually and analytically in line with prior research and developmental theory. Both early risk factors (e.g., peer delinquency and peer support for aggression) and promotive factors (e.g., prosocial behaviors) have been found to continue or increase across adolescence (Evans et al. 2016; Catalano et al. 2010; Malti et al. 2015; Padilla‐Walker et al. 2018). However, little longitudinal research has focused on some promotive factors (e.g., peer messages supporting nonviolence), though there is some evidence that prosocial peer selection is positively reinforcing (e.g., adolescents who are treated well by a peer or feel safe with a peer will spend more time with that peer) (Farrell et al. 2010a; Farrell et al. 2010b; Farrell et al. 2022).

In the initial models (before considering multiple groups by sex and grade), cross‐lagged findings showed that having been shot at and having seen someone carrying a gun or knife predicted increased peer support for aggressive responses to conflict 6 months later; and hearing gunfire when in or near home or school and having seen someone be shot predicted higher reports of peer delinquency across this same time period. Prior research primarily has focused on exposure associations with individual‐level factors and consistently has shown that adolescent exposure to firearms and firearm violence was associated with the adolescents’ own future engagement in delinquent behaviors (Bancalari et al. 2022; McGee et al. 2017; Quimby et al. 2018). The findings in this study revealed a relatively unexplored exposure path and are in line with a prior study that explored the directional path from a composite measure (combining violent victimization and witnessing) of community violence exposure to affiliated peer delinquency. Specifically, the prior study showed that Black middle‐school aged adolescents with a history of witnessing community violence reported higher rates of peer delinquency across time (Farrell et al. 2022). While peer delinquency and support for aggressive responses to conflict should not be conflated, they are correlated and predictive of one another across time (Lansford et al. 2020). The current study findings added to the literature by showing the associations between specific types of firearm exposure and increased peer engagement in delinquency and support for aggression over time.

There were only two promotive cross‐lagged findings which indicated that peer support for nonviolent responses to conflict was related to lower reports of having been shot at or having seen someone carrying a gun or knife 6 months later. Prior tests of relations between similar variables have found that peer support for nonviolent responses to conflict had a protective effect on the relation between community violence victimization and physical aggression, though this did not hold true for the relation between witnessing community violence and physical aggression (Coleman and Farrell 2021). Additionally, promotive effects between peer support for nonviolent responses to conflict and community violence victimization were not previously tested. The promotive findings in this study are unique and contribute to our understanding of positive peer influences that may be able to be bolstered to attenuate future exposure to firearm violence during early adolescence. These and the prior mentioned risk paths are discussed further in the context of sex and grade differences below.

### 4.2 Theoretical Implications and Developmental Considerations

The bidirectional and developmentally specific findings from the current study align with key tenets of the SDM (Catalano et al. 2021), which integrates principles of social learning theory (Bandura 1977) and social bond theory (Hirschi 1968). The SDM proposes that adolescents are socialized through dual pathways: one promotive (characterized by strong connections to prosocial peers and institutions) and one risk‐laden (characterized by weak bonds and reinforcement for antisocial behaviors). These pathways are shaped by the behaviors and norms modeled, reinforced, and endorsed within adolescents’ peer groups. The current study's findings, particularly those showing that exposure to firearm violence predicted greater peer support for aggression and delinquency, and that peer support for nonviolence predicted decreased exposure, reflect the dynamic and reciprocal nature of these processes.

Developmental theory also suggests that peer influence is not uniform across adolescence, but varies by age and context. During early adolescence, peers become increasingly salient as youth seek autonomy from adults and begin to solidify social identities (Huston and Ripke 2006). However, younger adolescents (e.g., 6th graders) may be more susceptible to direct peer modeling due to still‐developing cognitive control and weaker internalized norms (Coie and Kupersmidt 1983). In contrast, older adolescents (e.g., 8th graders) may experience intensified social pressures as peer hierarchies become more defined and expectations around toughness or loyalty become more entrenched, especially in high‐violence, urban settings. This may help explain why some cross‐lagged paths (e.g., from peer risk to later exposure, or from exposure to increased risk affiliations) were more pronounced among older students.

Sex differences in the observed pathways may also reflect differential engagement with the peer context. Prior work suggests that female adolescents may affiliate with peers who exhibit aggressive or delinquent behaviors not out of shared behavioral intentions, but as a form of social protection or perceived safety in environments marked by high threat (Mitchell et al. 2019). In contrast, male adolescents may be more likely to both influence and be influenced by peers through co‐offending or shared behavioral norms related to violence. These interpretations align with both the SDM and gendered socialization processes observed in the broader violence prevention literature.

Taken together, the findings support the importance of assessing not only risk but promotive peer factors, such as peer endorsement of nonviolent responses, which may serve as a buffer against exposure to community firearm violence. The fact that promotive influences were most evident in younger adolescents and females reinforces developmental and contextual models of resilience, and highlights the need for tailored, developmentally‐sensitive prevention strategies that account for variation in how adolescents engage with their peer ecologies over time.

### 4.3 Understanding Cross‐Lagged Associations by Sex and Grade

Results from the multiple group models showed more associations between exposure to community firearm violence and peer risk factors than with promotive factors, and that broadly, but not exclusively, when there were grade or sex differences in risk paths, these favored older (either 8th grade and not 6th or 7th grade or 7th and 8th graders and not 6th grade) students and females (rather than male or younger adolescents). The risk factors varied across the five exposure item models, though several patterns of results emerged by sex and grade.

#### 4.3.1 Cross‐Lagged Risk Effects by Sex

Female early adolescents’ exposure to community firearm violence predicted affiliations with peers who engaged in delinquent behavior and supported aggressive responses to potential conflict situations across the 6‐month time frame. These results encompassed outcomes including victimization (been shot at) and witnessing violence (seen someone carrying a gun or knife, heard gunfire when in or near school). The findings suggested that early adolescent females’ exposure to community firearm violence in a community context characterized by high levels of violence exposure influenced future peer affiliations. Prior studies of exposure to firearm violence in urban communities have found that female adolescents who witnessed indirect firearm violence (e.g., heard gunshots) reported higher rates of extreme distress, specifically fear, as a result (Mitchell et al. 2019). As a consequence of this exposure, it is possible that female adolescents may choose to align themselves with peers who make them feel safe or protected through behavior typically categorized as delinquent or supportive of aggressive behavior.

Additional results from the present study supported the premise that female adolescents, aligning themselves with peers who engage in delinquent behavior or support aggressive responses as a means of self‐preservation or safety, represents a double‐edged sword. Specifically, the findings showed that for female adolescents, peer support for aggressive responses to conflict was associated with increased frequency of hearing gunfire in or near home. While aligning themselves with peers who engage in delinquency or support aggression may increase their feeling of safety, the presence of peer conflict and the use of aggression to solve conflicts may put them in closer proximity to firearm behavior including gunfire (Mitchell et al. 2019; Oliphant et al. 2019; Schmidt et al. 2019).

Though there were fewer significant cross‐lagged paths for male adolescents, peer delinquency and peer support for aggressive responses to conflict increased male adolescents’ likelihood of severe victimization (been shot at) and witnessing violence (seen someone else be shot at) 6 months later. Prior findings have indicated that peer delinquent behavior is a stronger predictor of future delinquent behavior, and that physical victimization occurs more frequently among male versus female adolescents (Piquero et al. 2005; Prinstein et al. 2001). These findings highlighted the concerning consequences for Black male adolescents living in urban communities characterized by high levels of violence, even over short intervals of time during middle school.

Considering paths from violence exposure to peer factors, male adolescents who “heard gunfire when in or near home” reported higher frequencies of peer delinquency 6 months later. This is, again, representative of the cyclical nature of peer behaviors and exposure to firearm violence, where in affiliation with peers who engage in delinquent behavior may put early adolescents in closer proximity to community firearm violence, and exposure to community firearm violence may increase affiliation with peers who engage in delinquency or support aggressive responses. Taken together, the current study findings, especially those that pertain to female adolescents, are a unique addition to the literature as prior research primarily focused on male adolescents and their peer relationships as they relate to firearm violence outcomes (Oliphant et al. 2019; Schmidt et al. 2019). Our findings showed that female and male early adolescents and their peers were distinctly affected by exposure to community firearm violence, requiring a particular focus on sex differences in these relations.

#### 4.3.2 Cross‐Lagged Risk Effects by Grade

Across victimization and witnessing items, among older (7th and 8th grade or 8th grade only) early adolescents firearm violence was related to increased affiliation with peers who engage in delinquent behavior or supported aggressive responses to conflict. Additionally, bidirectional relations were identified for older early adolescents who reported having peers who supported aggressive responses to conflict and delinquent behavior led to increased witnessed violence (i.e., hearing gunfire in or near their home) and victimization (i.e., seeing someone be shot at). These findings speak to prior literature which suggested that early exposure to community violence and victimization increased likelihood for future delinquent peer affiliation (Burnside et al. 2018). Similarly, the presence of peer delinquency and support for aggressive responses may put early adolescents in closer proximity to firearm behavior including gunfire close to home.

Fewer cross‐lagged paths favored younger adolescents (6th graders or 6th and 7th graders), though exposure to community firearm victimization (i.e., been shot at) and witnessing violence (i.e., seen someone carrying a gun or knife; heard gunfire in or near home) predicted affiliation with adolescent peers who engaged in delinquent behaviors 6 months later. Interestingly, in one case, older adolescents (7th and 8th graders but not 6th graders) and younger adolescents (6th and 7th graders but not 8th graders) who reported having been shot at T1 increased rates of peer delinquent behavior at T2. One potential explanation for this grade‐level finding is that changes in peer affiliations are the result of the extreme deleterious consequences of having been shot at with a firearm in middle school, and the cascade of adverse relationships and experiences that have been found to follow seeing or experiencing a traumatic physical violence event (Holloway et al. 2023). For the last finding, 7th grade may be a pivotal point following the transition into 6th grade and the restructuring of peer groups from different elementary schools and before the 8th grade transition from middle to high school (McMillan and Freelin 2023).

These findings are consistent with prior research on risk associations for and negative outcomes from exposure to community violence and firearm behavior (Bancalari et al. 2022; Oliphant et al. 2019; Schmidt et al. 2019). Black male adolescents who are older have consistently higher rates of risk for exposure to community violence. In particular adolescents who live in urban areas with higher rates of community violence are disproportionately exposed to witnessing violence and violent victimization involving firearms, and as a result affiliated with peers who engaged in delinquent behaviors or support aggressive responses may be seen as a means of keeping oneself safe and adhering to social norms (Berkowitz 2002; Huesmann 2018). Additionally, the effect of violence exposure on peer affiliation for early adolescents reinforces that middle school, as a whole, is a critical period where violence exposure and peer affiliations are related for Black adolescents, and that researchers and interventionists may benefit from developing further understanding of malleable factors to target during this developmental period.

#### 4.3.3 Promotive Cross‐Lagged Effects by Sex and Grade

Promotive effects by sex and grade were seen for two of the five exposure items (i.e., “been shot at” and “seen someone carrying a gun or knife”). Eighth graders but not 6th and 7th graders reported that having peers who supported nonviolent responses to conflict and engaged in prosocial behaviors predicted a lower likelihood of being shot at 6 months later. For 6th and 7th but not 8th graders and for female adolescents, peer support for nonviolent responses to conflict was associated with a lower likelihood of seeing someone carrying a gun or knife. In prior literature, Black adolescents who reported having prosocial peers in middle school also reported higher rates of self‐esteem and school connectedness (Quimby et al. 2018), both of which are factors linked with lower rates of adolescents’ own aggression and victimization. As firearm violence typically occurs among peers who know one another, adolescents whose peers are not engaged in violence, or who promote nonviolence, are less likely to be exposed to firearm violence themselves (Jung and Schröder‐Abé 2019; Oliphant et al. 2019). While this study found sex differences in the strength of relations between paths, prior studies have not consistently found that adolescents’ biological sex plays a role in the associations of peer behaviors with adolescent outcomes, even in similar socio‐demographic samples (Quimby et al. 2018). Additionally, prior studies have not investigated differences by middle school grade, adding to the uniqueness of these findings. Altogether, the current study findings indicated a strong interplay between peer behavior and middle‐school adolescents’ exposure to firearm violence in their community (Oliphant et al. 2019).

### 4.4 Limitations

This study has several limitations related to the secondary data approach and methodology. This study reflected a limitation of the broader community firearm violence exposure literature in its use of single‐item measures of the construct. There is likely additional knowledge to be garnered from a robust measure of exposure to community firearm violence that cannot be known through item‐level analyses. However, because the current study used five individual items that were representative of the larger construct, rather than one or two items as seen in prior studies (Bancalari et al. 2022), it provided a more robust understanding of peer risk and promotive factors associated with this construct. The fact that different patterns of findings emerged across these indicators of firearm violence exposure suggests there was a benefit in testing the exposure items independently. Additionally, participants’ perception of their peers’ behavior may be limited and reflect personal biases based on their own engagement (or lack thereof) in delinquent and prosocial behavior, as well as the extent of their knowledge of their peers’ behavior. Social Norms Theory posited that adolescents often have misperceptions of their peer's behavior, and as such peer reports of their own behavior, or adolescents’ self‐report of their own prosocial or delinquent behavior may be important in contextualizing the current study findings (Berkowitz 2002). Questions pertaining to adolescents’ experiences with community firearm violence were limited to rates of exposure in the prior 3 months and did not address the specificity, context, or social networks (e.g., how close in proximity were adolescents to the exposure and did it involve someone they knew or were close to; Gill and Fox 2022).

Further, conclusions about generalizability in this study are finite. A key study strength is the relevant sample of Black adolescents who live in disinvested urban communities where rates of violence are disproportionately high, making the research questions and analytic design appropriate for use in this sample. However, the findings of the present study also speak to the experiences of the early adolescents in this specific sample and may not be generalizable to adolescents living in other social or ecological contexts, social and political climates, or from other racial and ethnic backgrounds. Additionally, our covariate selection was guided by theoretical and methodological considerations to avoid overadjustment and potential collider bias. As such, we did not include certain baseline measures (e.g., mental health, substance use, prior victimization), which, while relevant to youth firearm violence, may act as mediators rather than confounders in our model. Additionally, some of these variables were not consistently available at baseline in our dataset, which limited our ability to include them without introducing measurement bias. Thus correlational nature of the study precludes any conclusions about causality, though the longitudinal design may clarify the temporal ordering of the examined constructs.

### 4.5 Future Directions

There is considerable work to be done to better understand early adolescents’ firearm‐related exposures. To build on prior literature and the current study, several steps may be taken. First, additional insight may be gained into the trajectory of relations and underlying mechanisms driving these relations by testing peer and exposure variables at more than two time points or across a greater period of time, as well as considering moderators and mediators of these relations. For example, adolescents’ own beliefs about fighting, threat appraisal, or prior exposure to violence may mediate longitudinal relations between exposure variables and peer factors. Prior research has indicated the potential interplay of multiple types of violence experiences (e.g., in‐person and cyber bullying, peer‐based or dating physical aggression or victimization) on firearm violence exposure and behavior (e.g., community violence victimization, firearm carrying), and thus it may be helpful to account for other subtypes of violence exposure across contexts in examining these relations. Finally, future research may benefit from conducting person‐centered analyses (e.g., a latent class analysis) to identify patterns of risk and protective factors related to firearm violence exposure.

In addition to advancing research, these findings offer direction for prevention and intervention efforts. Middle school appears to be a particularly salient period for shaping peer affiliations and exposure to firearm violence, with differences emerging by both grade and sex. Prevention programs may benefit from emphasizing promotive peer factors such as nonviolent conflict responses and prosocial behaviors, particularly in earlier grades (e.g., 6th and 7th), when such influences were associated with reduced future exposure. For older youth, particularly 8th graders and females, programming may need to address the complex reasons behind affiliating with delinquent or aggressive peers, including perceived safety or social survival. Intervening early, before peer affiliations solidify and exposure escalates, may be key in disrupting the cyclical nature of violence exposure and risky peer influence during this developmental window.

### 4.6 Conclusion

This study used a developmentally informed, prevention‐oriented lens to explore the extent to which peer risk and promotive factors were bidirectionally associated with five different items assessing exposure to community firearm violence while controlling for a robust set of household demographic factors in a sample of Black middle school aged adolescents from disinvested urban communities. Across the five models, all peer risk and promotive factors and exposure to community firearm violence items showed stability across a 6‐month period. More differences were seen in associations between exposure items and risk factors than with promotive factors, and risk paths favored older early adolescents and males. It is important to note that exposure to community firearm violence for Black adolescents and families in these communities is a symptom of greater socio‐structural influences, which were not accounted for in this dataset. Despite limitations to the study design, the longitudinal nature of this study, as well as the use of promotive in addition to peer risk factors in a contextually and demographically relevant sample, allows for important insight into factors that can be targeted for the prevention of community firearm violence exposure in urban communities.

## Author Contributions

**Colleen S. Walsh:** conceptualized, conducted analyses, drafted the document and tables, and edited the manuscript based on coauthor feedback. **Terri N. Sullivan:** procured funding, supported conceptualization, and edited the manuscript. **Wendy L. Kliewer:** supported conceptualization, and edited the manuscript.

## Ethics Statement

The study was approved by the university institutional review board.

## Consent

At the time of data collection, participants provided written parental consent and informed youth assent.

## Conflicts of Interest

The authors declare no conflicts of interest.

## Supporting information


**Supplementary Table 1:** *Correlations between all Study Variables (N* = 479*)*. **Supplemental Table 2:** *Summary of Cross‐lagged Findings*.

## Data Availability

The data that support the findings of this study are available on request from the corresponding author. The data are not publicly available due to privacy or ethical restrictions.
