# Supplementary material for: Longitudinal Associations Between Peer Risk and Promotive Factors and Exposure to Community Firearm Violence Among Black Adolescents
Source: J Community Psychol. 2025 Oct 10;53(8):e70047. doi: 10.1002/jcop.70047 (PMC12513415; doi:10.1002/jcop.70047)
Supplement: Supplementary file 1 — Supplementary Table 1: Correlations between all Study Variables (N = 479). Supplemental Table 2: Summary of Cross‐lagged Findings. [file JCOP-53-0-s001.docx]

| **Supplementary Table 1.** | | | | | | | | | | | | | | | | | | | | | | | | | |  |
| --- | --- | --- | --- | --- | --- | --- | --- | --- | --- | --- | --- | --- | --- | --- | --- | --- | --- | --- | --- | --- | --- | --- | --- | --- | --- | --- |
| *Correlations between all Study Variables (N* = 479*)* | | | | | | | | | | | | | | | | | | | | | | | | | |  |
| Variable | | 1 | 2 | | 3 | 4 | | 5 | | 6 | | 7 | | 8 | | 9 | | 10 | | 11 | | 12 | 13 | |  |  |
| 1. T1 “actually been shot at with a gun?” | - | | |  |  | |  | |  | |  | |  | |  | |  | |  | |  |  | |  | | |
| 1. T1 “actually seen someone carrying a gun or knife” | .25** | | | - |  | |  | |  | |  | |  | |  | |  | |  | |  |  | |  | | |
| 1. T1 “heard gunfire outside when you were in or near your home” | .22^**^ | | | .50^**^ | - | |  | |  | |  | |  | |  | |  | |  | |  |  | |  | | |
| 1. T1 “heard gunfire outside when you were in or near your school building” | .25^**^ | | | .39^**^ | .32^**^ | | - | |  | |  | |  | |  | |  | |  | |  |  | |  | | |
| 1. T1 “seen someone else be shot with a gun” | .26^**^ | | | .42^**^ | .38^**^ | | .26^**^ | | - | |  | |  | |  | |  | |  | |  |  | |  | | |
| 1. T1 Peer Delinquent Behavior | .26^**^ | | | .30^**^ | .17^**^ | | .11^*^ | | .22^**^ | | - | |  | |  | |  | |  | |  |  | |  | | |
| 1. T1 Peer Prosocial Behavior | .01 | | | -.13^**^ | .01 | | -.07 | | -.08 | | -.13^**^ | | - | |  | |  | |  | |  |  | |  | | |
| 1. T1 Peer Support for Aggression | -.004 | | | .14^**^ | .12^*^ | | .04 | | .12^*^ | | .21^**^ | | -.34^**^ | | - | |  | |  | |  |  | |  | | |
| 1. T1 Peer Support for Nonviolence | .001 | | | -.15^**^ | -.11^*^ | | -.06 | | -.04 | | -.23^**^ | | .37^**^ | | -.60^**^ | | - | |  | |  |  | |  | | |
| 1. T2 “actually been shot at with a gun?” | **.37^**^** | | | .29^**^ | .23^**^ | | .03 | | .21^**^ | | .23^**^ | | -.09 | | .03 | | -.13^*^ | | - | |  |  | |  | | |
| 1. T2 “actually seen someone carrying a gun or knife” | .13^*^ | | | **.45^**^** | .31^**^ | | .19^**^ | | .33^**^ | | .17^**^ | | -.14^**^ | | .13^*^ | | -.19^**^ | | .29^**^ | | - |  | |  | | |
| 1. T2 “heard gunfire outside when you were in or near your home” | .17^**^ | | | .41^**^ | **.48^**^** | | .20^**^ | | .34^**^ | | .13^*^ | | -.03 | | .14^*^ | | -.13^*^ | | .27^**^ | | .46^**^ | - | |  | | |
| 1. T2 “heard gunfire outside when you were in or near your school building” | .25^**^ | | | .22^**^ | .19^**^ | | **.17^**^** | | .23^**^ | | .08 | | .03 | | .06 | | -.06 | | .28^**^ | | .36^**^ | .32^**^ | | - | | |
| 1. T2 “seen someone else be shot with a gun” | .23^**^ | | | .41^**^ | .32^**^ | | .20^**^ | | **.46^**^** | | .10 | | .004 | | .004 | | -.003 | | .41^**^ | | .52^**^ | .35^**^ | | .28^**^ | | |
| 1. T2 Peer Delinquent Behavior | .17^**^ | | | .16^**^ | .15^**^ | | .13^*^ | | .20^**^ | | **.35^**^** | | -.10 | | .13^*^ | | -.14^*^ | | .34^**^ | | .23^**^ | .20^**^ | | .14^**^ | | |
| 1. T2 Peer Prosocial Behavior | .08 | | | -.05 | .003 | | -.07 | | .004 | | -.08 | | **.44^**^** | | -.30^**^ | | .28^**^ | | .01 | | .001 | .01 | | .01 | | |
| 1. T2 Peer Support for Aggression | .06 | | | .23^**^ | .13^*^ | | .02 | | .17^**^ | | .13^*^ | | -.27^**^ | | **.57^**^** | | -.51^**^ | | .04 | | .12^*^ | .12^*^ | | .06 | | |
| 1. T2 Peer Support for Nonviolence | .04 | | | -.06 | -.02 | | .01 | | -.02 | | -.09 | | .24^**^ | | -.44^**^ | | **.58^**^** | | -.02 | | -.11 | -.10 | | .05 | | |
| Covariates |  | | |  |  | |  | |  | |  | |  | |  | |  | |  | |  |  | |  | | |
| 1. Biological Sex (male) | .14^**^ | | | .07 | -.004 | | .10^*^ | | .04 | | .11^*^ | | -.20^**^ | | .08 | | -.16^**^ | | .13^*^ | | .07 | -.02 | | .07 | | |
| 1. Intervention Condition | .05 | | | -.05 | -.03 | | .01 | | .00 | | .01 | | -.01 | | .09 | | -.01 | | .11^*^ | | -.01 | .07 | | .09 | | |
| 1. Grade – 6^th^ | .03 | | | .01 | .11^*^ | | .03 | | .05 | | -.10^*^ | | .18^**^ | | -.11^*^ | | .16^**^ | | .07 | | .02 | .14^**^ | | .03 | | |
| 1. Grade – 7^th^ | -.03 | | | .02 | .002 | | -.04 | | -.04 | | -.01 | | -.08 | | .03 | | -.01 | | -.09 | | -.04 | .01 | | -.01 | | |
| 1. Grade – 8^th^ | .002 | | | -.03 | -.12^*^ | | .01 | | -.02 | | .11^*^ | | -.11^*^ | | .09 | | -.15^**^ | | .02 | | .02 | -.15^**^ | | -.03 | | |
| 1. T1 Two caregiver household | .001 | | | .03 | .04 | | .05 | | .04 | | -.02 | | .07 | | -.13^**^ | | .10^*^ | | .004 | | .05 | .08 | | .06 | | |
| 1. T1 One caregiver household | -.002 | | | -.04 | -.07 | | -.04 | | -.08 | | .02 | | -.06 | | .10^*^ | | -.08 | | -.08 | | -.03 | -.11^*^ | | -.08 | | |
| 1. T1 Father figure present in household | -.08 | | | .02 | -.05 | | .02 | | -.001 | | -.04 | | .03 | | -.06 | | .03 | | -.02 | | .04 | .06 | | .05 | | |
| 1. T2 Two caregiver household | -.03 | | | -.06 | -.04 | | -.05 | | -.03 | | -.02 | | .14^**^ | | -.08 | | .05 | | .05 | | .02 | .09 | | -.01 | | |
| 1. T2 One caregiver household | -.02 | | | -.01 | -.01 | | .04 | | -.01 | | -.03 | | -.03 | | .12^*^ | | -.04 | | -.05 | | .003 | -.04 | | -.03 | | |
| 1. T2 Father figure present in household | -.08 | | | -.06 | -.08 | | -.02 | | -.04 | | -.07 | | .11^*^ | | -.06 | | .07 | | .01 | | .02 | .05 | | -.002 | | |
| *Note.* **p*<.05, ***p*<.01. T1 = Time 1 (Fall); T2 = Time 2 (Spring). Stability correlations are bolded. | | | | | | | | | | | | | | | | | | | | | | | | | |  |

| **Supplementary Table 1 (continued)** | | | | | | | | | | | | | | | | | | | |
| --- | --- | --- | --- | --- | --- | --- | --- | --- | --- | --- | --- | --- | --- | --- | --- | --- | --- | --- | --- |
| *Correlations between all Study Variables (N* = 479*)* | | | | | | | | | | | | | | | | | | | |
| Variable | 14 | 15 | 16 | | | | | 17 | 18 | | 19 | 20 | 21 | 22 | 23 | 24 | 25 | 26 |  |
| 1. T2 “seen someone else be shot with a gun” | - |  | | |  |  | | | |  |  |  |  |  |  |  |  |  |  |
| 1. T2 Peer Delinquent Behavior | .334^**^ | - | | |  |  | | | |  |  |  |  |  |  |  |  |  |  |
| 1. T2 Peer Prosocial Behavior | .026 | .018 | | | - |  | | | |  |  |  |  |  |  |  |  |  |  |
| 1. T2 Peer Support for Aggression | .050 | .095 | | -.374^**^ | | - | | | |  |  |  |  |  |  |  |  |  |  |
| 1. T2 Peer Support for Nonviolence | .003 | -.18^**^ | | | .39^**^ | -.60^**^ | | | | - |  |  |  |  |  |  |  |  |  |
| Covariates |  |  | | |  |  | | | |  |  |  |  |  |  |  |  |  |  |
| 1. Biological Sex | .05 | .06 | | | -.19^**^ | .14^*^ | | | | -.13^*^ | - |  |  |  |  |  |  |  |  |
| 1. Intervention Condition | .001 | .07 | | | .03 | -.001 | | | | .11^*^ | .04 | - |  |  |  |  |  |  |  |
| 1. Grade – 6^th^ | .08 | -.04 | | | .09 | -.05 | | | | .10 | -.01 | .004 | - |  |  |  |  |  |  |
| 1. Grade – 7^th^ | -.04 | -.05 | | | -.11^*^ | -.05 | | | | .00 | -.02 | .01 | -.51^**^ | - |  |  |  |  |  |
| 1. Grade – 8^th^ | -.05 | .09 | | | .02 | .10 | | | | -.10 | .03 | -.02 | -.51^**^ | -.49^**^ | - |  |  |  |  |
| 1. T1 Two caregiver household | .07 | -.04 | | | .08 | -.08 | | | | .10 | -.05 | .04 | -.01 | .05 | -.03 | - |  |  |  |
| 1. T1 One caregiver household | -.12^*^ | .03 | | | -.01 | .01 | | | | -.07 | .004 | -.07 | -.03 | -.07 | .10^*^ | -.84^**^ | - |  |  |
| 1. T1 Father figure present in household | .06 | -.07 | | | .06 | -.08 | | | | .06 | -.001 | .04 | -.02 | .05 | -.03 | .71^**^ | -.58^**^ | - |  |
| 1. T2 Two caregiver household | .06 | -.04 | | | .04 | -.01 | | | | .01 | -.01 | .05 | -.01 | .07 | -.05 | .46^**^ | -.47^**^ | .39^**^ |  |
| 1. T2 One caregiver household | -.06 | .03 | | | .003 | .02 | | | | -.04 | .001 | -.07 | -.01 | -.02 | .03 | -.47^**^ | .52^**^ | -.39^**^ |  |
| 1. T2 Father figure present in household | .06 | -.04 | | | .03 | | -.06 | | | .04 | .03 | .06 | .003 | .03 | -.03 | .37^**^ | -.39^**^ | .58^**^ |  |
| *Note.* **p*<.05, ***p*<.01. T1 = Time 1 (Fall); T2 = Time 2 (Spring). | | | | | | | | | | | | | | | | | | | |

| **Supplementary Table 1 (continued)** | | | | | | | | | | | | | | |
| --- | --- | --- | --- | --- | --- | --- | --- | --- | --- | --- | --- | --- | --- | --- |
| *Correlations between all Study Variables (N* = 479*)* | | | | | | | | | | | | | | |
| Variable | 27 | 28 | 29 |  |  |  |  |  |  |  |  |  |  |  |
| Covariates |  |  |  |  |  |  |  |  |  |  |  |  |  |  |
| 1. T2 Two caregiver household | - |  |  |  |  |  |  |  |  |  |  |  |  |  |
| 1. T2 One caregiver household | -.66^**^ | - |  |  |  |  |  |  |  |  |  |  |  |  |
| 1. T2 Father figure present in household | .72^**^ | -.43^**^ | - |  |  |  |  |  |  |  |  |  |  |  |
| *Note.* **p*<.05, ***p*<.01. T1 = Time 1 (Fall); T2 = Time 2 (Spring). | | | | | | | | | | | | | | |

| **Supplemental Table 2.** | | | | | |
| --- | --- | --- | --- | --- | --- |
| *Summary of Cross-lagged Findings* | | | | | |
| **Exposure Item (past 3 months)** | **“been shot at”** | **“seen someone carrying a gun or knife”** | **“heard gunfire when in or near home”** | **“heard gunfire when in or near school”** | **“seen someone be shot at”** |
| **Peer factors** | T1 “been shot at” →↑ T2 peer support for aggression  T1 peer support for nonviolence →↓ T2 “been shot at” | T1 “seen someone carrying a gun or knife” →↑ T2 peer support for aggression  T1 peer support for nonviolence →↓ T2 “seen someone carrying a gun or knife” | T1 “heard gunfire when in or near home” →↑ T2 Peer delinquency | T1 “heard gunfire when in or near school” →↑ T2 Peer delinquency | T1 “seen someone be shot at” →↑ T2 Peer delinquency |
| **Cross-lagged findings by sex** | **Females:** T1 “been shot at” →↑ T2 Peer delinquency  **Males**: T1 Peer delinquency →↑ T2 “been shot at” | **Females**: T1“seen someone carrying a gun or knife” →↑ T2 Peer delinquency  **Females**: T1 “seen someone carrying a gun or knife” →↑ T2 Peer support for aggression  **Females**: T1 Peer support for nonviolence →↓ T2 “seen someone carrying a gun or knife” | **Females**: T1 Peer support for aggression →↑ T2 “heard gunfire when in or near home”  **Males**: T1“heard gunfire when in or near home” →↑ T2 Peer delinquency | **Female**: T1 “heard gunfire when in or near school” →↑ T2 Peer delinquency | **Males**: T1 Peer support for aggression →↑ T2 “seen someone be shot at” |
| **Cross-lagged findings by grade** | **6/7th**: Peer delinquency →↑ T2 “been shot at”  **7th/8th**: T1 “been shot at” →↑ T2 Peer delinquency  **6/7th**: T1 “been shot at” →↑ T2 Peer delinquency  **8th**: T1 Peer support for nonviolence → ↓ T2 “been shot at”  **8th:** T1 “been shot at” →↑ T2 Peer support for aggression  **8th**: T1 Peer prosocial behavior → ↓ T2 “been shot at” | **6/7th**: T1“seen someone carrying a gun or knife” →↑ T2 Peer delinquency  **7/8th**: T1 “seen someone carrying a gun or knife” →↑ T2 Peer support for aggression  **6th**: T1 Peer support for nonviolence →↓ T2 “seen someone carrying a gun or knife”  **6/7th**: T1 peer support for nonviolence →↓ T2 “seen someone carrying a gun or knife” | **6th**: T1“heard gunfire when in or near home” →↑ T2 Peer delinquency  **6/7th**: T1 “heard gunfire when in or near home” →↑ T2 Peer delinquency  **8th**: T1 Peer support for aggression →↑ T2 “heard gunfire when in or near home”  **8th**: T1 “heard gunfire when in or near home” →↑ T2 Peer support for aggression | **7th/8th**: T1 “heard gunfire when in or near school” →↑ T2 Peer delinquency  **7th/8th**: T1 “heard gunfire when in or near school” →↑ T2 Peer support for nonviolence | **7/8th:** T1 Peer Delinquent behavior →↑ T2 “seen someone be shot at”  **7/8th**: T1 “seen someone be shot at” →↑ T2 Peer delinquency  **8th**: T1 “seen someone be shot at” →↑ T2 Peer delinquency  **7/8th**: T1 “seen someone be shot at” →↑ T2 Peer support for aggression  **8th**: T1 “seen someone be shot at” →↑ T2 Peer support for aggression |
| *Note*. ↑ = increase; → = relation; ↓ = decrease. | | | | | |
